# Retraction Note: Phyto-assisted synthesis of zinc oxide nanoparticles using *Bauhinia* *variegata* buds extract and evaluation of their multi-faceted biological potentials

**DOI:** 10.1038/s41598-025-03834-7

**Published:** 2025-06-04

**Authors:** Sehrish Arafat, Javed Iqbal, Banzeer Ahsan Abbasi, Shumaila Ijaz, Tabassum Yaseen, Ghulam Murtaza, Rafi Ullah, Farishta Zarshan, Zakir Ullah, Zulfiqar Ali Sahito, Saeedah Musaed Almutairi, Mohamed S. Elshikh, Saltanat Aghayeva, Muhammad Rizwan, Rashid Iqbal

**Affiliations:** 1 Department of Botany, Bacha Khan University, Charsadda, 24420 Khyber Pakhtunkhwa Pakistan; 2 Department of Botany, Rawalpindi Women University, 6th Road, Satellite Town, Rawalpindi, 46300 Pakistan; 3 School of Biomedical Engineering, Shenzhen University Medical School, Shenzhen University, Shenzhen, 518060 People’s Republic of China; 4 School of Agriculture, Yunnan University, Kunming, Yunnan, 650504 People’s Republic of China; 5 Department of Plant Sciences, Faculty of Biological Sciences, Quaid-i-Azam University, Islamabad, 45320 Pakistan; 6 Ministry of Education (MOE) Key Laboratory of Environmental Remediation and Ecosystem Health, College of Environmental and Resources Science, Zhejiang University, Hangzhou, 310058 People’s Republic of China; 7 Department of Botany and Microbiology, College of Science, King Saud University, P.O. 2455, 11451 Riyadh, Saudi Arabia; 8 Department of Life Sciences, Western Caspian University, Baku, Azerbaijan; 9 Institute of Crop Science and Resource Conservation (INRES), University of Bonn, 53115 Bonn, Germany; 10 Department of Agronomy, Faculty of Agriculture and Environment, The Islamia University of Bahawalpur, Bahawalpur, 63100 Pakistan

Retraction of: *Scientific Reports* 10.1038/s41598-024-72250-0, published online 11 September 2024

The Editors have retracted this Article.

After publication of this Article, concerns were raised regarding some image irregularities and similarities across publications. Specifically:

- The XRD spectra in Figure 4 appear to have unusual highly similar noise and peak patterns within the same spectra.
- There appear to be high image similarity across publications between the SEM images of Zinc oxide nanoparticles (ZnONPs) in Figure 6 and nickel oxide nanoparticles (NiONPs) in Figure 5A-B in [1].

The Authors did not respond to request for the data and explanations. The Editors no longer have confidence in the data presented in this Article.

None of the Authors responded to the Editors’ correspondence about this retraction.
